# The CT delta-radiomics based machine learning approach in evaluating multiple primary lung adenocarcinoma

**DOI:** 10.1186/s12885-022-10036-1

**Published:** 2022-09-03

**Authors:** Yanqing Ma, Jie Li, Xiren Xu, Yang Zhang, Yi Lin

**Affiliations:** Department of Radiology, Cancer Center, Zhejiang Provincial People’s Hospital, Affiliated People’s Hospital, Hangzhou Medical College, Hangzhou, Zhejiang China

**Keywords:** Lung, Adenocarcinoma, Multiple, Computed tomography, Radiomics

## Abstract

**Object:**

To evaluate the difference between multiple primary lung adenocarcinoma (MPLA) and solitary primary lung adenocarcinoma (SPLA) by delta-radiomics based machine learning algorithms in CT images.

**Methods:**

A total of 1094 patients containing 268 MPLAs and 826 SPLAs were recruited for this retrospective study between 2014 to 2020. After the segmentation of volume of interest, the radiomic features were automatically calculated. The patients were categorized into the training set and testing set by a random proportion of 7:3. After feature selection, the relevant classifiers were constructed by the machine learning algorithms of Bayes, forest, k-nearest neighbor, logistic regression, support vector machine, and decision tree. The relative standard deviation (RSD) was calculated and the classification model with minimal RSD was chosen for delta-radiomics analysis to explore the variation of tumor during follow-up surveillance in the cohort of 225 MPLAs and 320 SPLAs. According to the different follow-up duration, it was divided into group A (3–12 months), group B (13–24 months), and group C (25–48 months). Then the corresponding delta-radiomics classifiers were developed to predict MPLAs. The area under the receiver operator characteristic curve (AUC) with 95% confidence interval (CI) was quantified to evaluate the efficiency of the model.

**Results:**

To radiomics analysis, the forest classifier (FC-radio) with the minimal RSD showed the better stability with AUCs of 0.840 (95%CI, 0.810–0.867) and 0.670 (95%CI, 0.611–0.724) in the training and testing set. The AUCs of the forest classifier based on delta-radiomics (FC-delta) were higher than those of FC-radio. In addition, with the extension of follow-up duration, the performance of FC-delta in Group C were the best with AUCs of 0.998 (95%CI, 0.993–1.000) in the training set and 0.853 (95%CI, 0.752–0.940) in the testing set.

**Conclusions:**

The machine-learning approach based on radiomics and delta-radiomics helped to differentiate SPLAs from MPLAs. The FC-delta with a longer follow-up duration could better distinguish between SPLAs and MPLAs.

**Supplementary Information:**

The online version contains supplementary material available at 10.1186/s12885-022-10036-1.

## Introduction

Lung carcinoma is the commonest cause of cancer-related death in China and is also a major health challenge worldwide [1]. The frequency and detection of lung carcinoma have descended gradually in the US, while they have remarkably increased in China over recent years [2]. Patients who survive one occurrence of non-small-cell lung carcinoma are at high risk of a second malignancy [3]. Cases of multiple primary lung carcinoma are increasing, mainly thanks to the improvement of diagnostic strategies, surveillance modalities, and the aging population [4]. The diagnostic criteria of multiple primary lung carcinomas was firstly established by Martini and Melamed in the year of 1975 [5] and was renewed by the American College and Chest Physicians (ACCP) in 2007 [6]. It is classified into synchronous phenotype when the second lung carcinoma was simultaneously diagnosed within 2 years after the primary lesion, and metachronous phenotype when it was separately diagnosed more than 2 years after the initial surgery [7]. Otherwise, when patients has only single primary lung carcinoma, it is defined as a solitary primary lung carcinoma. Duchateau et al. firstly indicated that 25% of patients accompanied with multiple primary lung carcinomas, and patients with and without those had different growth habits [8].

Adenocarcinoma has become the most prevalent sub-type of lung cancer. Therefore Evaluating the multiple primary lung adenocarcinomas (MPLAs) from solitary primary lung adenocarcinomas (SPLAs) by radiological methods is of great significance, while the conventional experience was difficulty in diagnosis [7]. Further analysis is therefore needed to comprehend more clearly. Radiomics converts the traditional radiological images into a large amount of minable high-dimensional data to explore the potential imaging biomarkers [9] and support decision making [10]. And the delta-radiomics is the change of radiomic features after treatment or surveillance [11]. Undoubtedly, a delta-radiomics approach will have a growing impact on distinction the MPLAs and SPLAs, which will enable optimized management of patients with MPLAs. To best of our knowledge, there is no study focused on the delta-radiomics difference between MPLAs and SPLAs. The purpose of our study is to evaluate the delta-radiomic influence of MPLAs on prognosis to help us better understand their difference with SPLAs.

## Materials and methods

This retrospective study was approved by the Institutional Review Board of Zhejiang Provinical People’s Hospital (NO. 2020QT108), which waived the informed consent of all patients.

### Patients screening

**Fig. 1:**
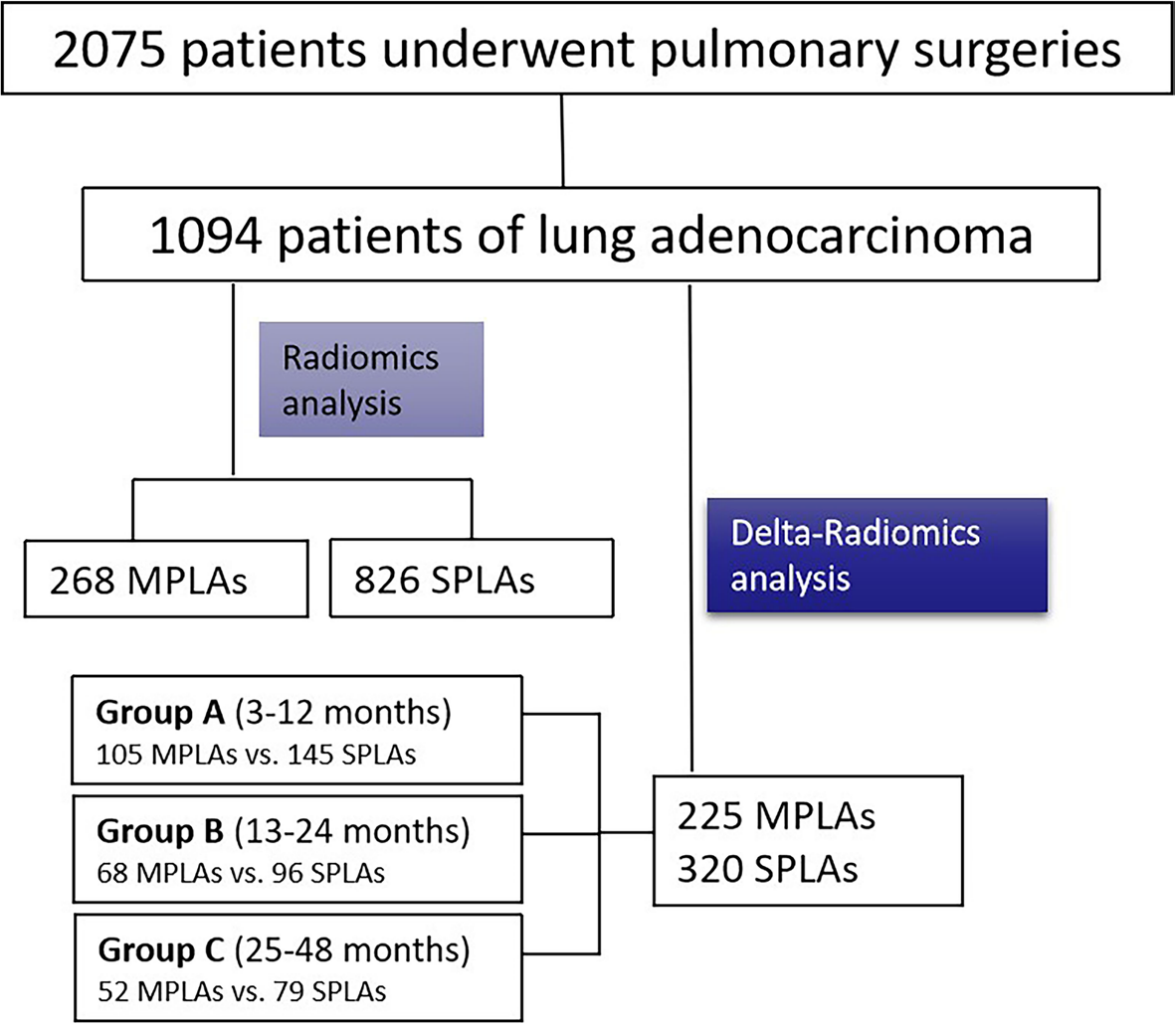
The flow diagram of patients selection

This retrospective study enrolled 1094 patients who were pathologically diagnosed as lung adenocarcinoma after 6 years follow-up surveillance, including 826 patients with SPLAs and 268 MPLAs, from January 2014 to December 2020. Among these patients, there were 320 SPLAs and 225 MPLAs patients with regular surveillance were incorporated for delta-radiomics analysis (Fig. 1). The inclusion criteria were as follows: (1) tumors were classified to be MPLAs according to the criteria of the 2^nd^ edition of ACCP evidence-based clinical practice guidelines [6] (Table 1); (2) patients had only one primary lung tumor at the time cut-off of inclusion were classified to be SPLAs; (3) patients were pathologically proved to be minimally invasive (MIA) or invasive adenocarcinoma (IAC) of lung; (4) patients underwent CT examinations with the same protocol. The exclusion criteria were as follows: (1) patients were pathologically confirmed to be atypical adenomatous hyperplasia, in situ adenocarcinoma, or pulmonary squamous carcinoma; (2) patients was pathologically confirmed by needle biopsy; (3) patients were treated with the methods of radiation, chemotherapy, or radio-chemotherapy.

**Table 1:** The criteria to diagnose multiple primary lung carcinoma according to ACCP

Multiple primary lung carcinomas
Same histology, anatomically separated
Carcinomas in different lobes
And no N2,3 involvement
And no systemic metastases
Same histology, temporally separated
≥ 4-yr interval between carcinomas
And no systemic metastases from either carcinoma
Different histology
Different histology type
Or different molecular genetic characteristics
Or arising separately from foci of carcinoma in situ

### CT examination and volume of interest segmentation

All the patients underwent CT unenhanced examinations in Somanton Definition AS 64 or 128 CT (Siemens Medical Solutions, Germany). The scan parameters were as follows: tube voltage, 120 kVp; tube current, 200 mA; rotation speed, 0.75 s; beam pitch, 1.375; pixel matrix, 512*512; detector collimation, 64*0.625 mm; slice thickness, 2.0 mm; reconstruction interval, 2.0 mm; width of lung window, 1500HU; level of lung window, -600HU.

**Fig. 2:**
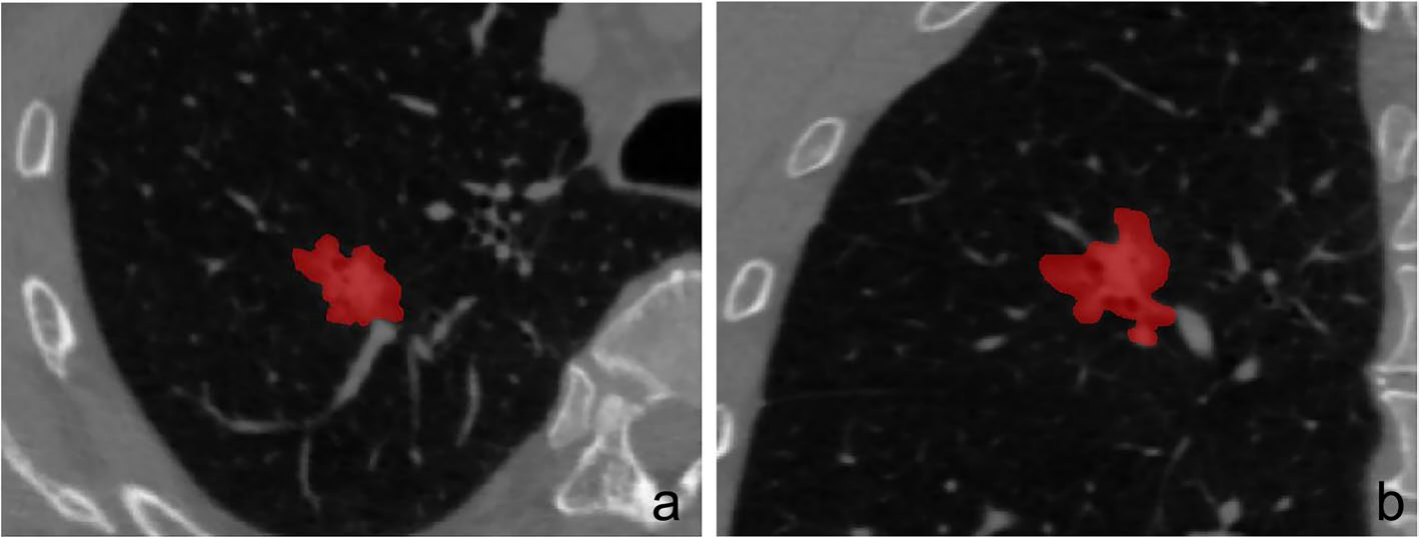
The VOI of tumor was manually depicted in the software of “ITK-snap”

The tumoral volume of interest (VOI) was depicted in software of “ITK-snap 3.8.0” (http://www.itksnap.org/pmwiki/) by two radiologists with 10 (Doctor Ma) and 12 years (Doctor Li) of experience, manually (Fig. 2a, b). Then, the radiomic features were automatically calculated in software of “A.K. 3.0.0” (GE Healthcare) after steps of preprocessing involved resampling images to be 1.0 mm at X/Y/Z space, reducing the image noise by a method of Gaussian, and discretizing the gray level to the range of 1 to 32. The intra-class correlation coefficients (ICCs) of radiomic features from two radiologists were calculated to evaluate the agreement between different observers. The radiomic features with ICCs greater than 0.75 were selected and the mean values of radiomic features from two radiologists were calculated for further analysis.

### Radiomics and delta-radiomics analysis

Prior to radiomic analysis, the steps of excluding variables with zero variance, replacing abnormal values by median values, and standardization were adopted to normalize radiomic features. Then the cohort was divided into the training set and testing set with a random proportion of 7:3. In order to eliminate the influence of unbalanced sample size, the way of synthetic minority over-sampling technique (SMOTE) was carried out [12]. After the methods of analysis of variance, correlation analysis with a threshold of 0.7, and gradient boosting decision tree (GBDT), the optimal radiomic features were extracted. Ultimately, the corresponding machine learning based classifiers including Bayes, forest, k-nearest neighbor, logistic regression, support vector machine, and decision tree algorithms were developed to identify MPLAs and SPLAs. The relative standard deviation (RSD) was calculated and the classification model with minimal RSD was chosen for further analysis. The area under the curve (AUC) with 95% confidence interval (95%CI) of receiver operator characteristic curve (ROC) was quantified to evaluate the efficiency of the machine learning based classifiers.

With consideration of the different progression during regular follow-up surveillance between MPLAs and SPLAs, the delta-radiomics was utilized. The delta-radiomics was defined as the change of radiomic features between baseline and follow-up surveillance, which was divided into three groups including Group A (3–12 months), Group B (13–24 months), and Group C (25–48 months), according to different follow-up surveillance. The equation of delta-radiomics was: (follow-up radiomics—baseline radiomics)/follow-up interval. The specific information of radiomics and delta-radiomics analysis were listed in Supplementary Material.

### Statistics

The general clinical characteristics were analysis by software of “SPSS 22.0” with methods of student’s t-test or chi-square test. The methods of radiomic feature selection including variance, correlation analysis, GBDT, and machine learning algorithms were performed by the software of “Python 3.5”. The ROC curve was delineated by the software of “MedCalc 15.8”. A *p*-value less than 0.05 indicates statistical significance.

## Results

### Patient’s general information

There were 1094 patients with 268 MPLAs and 826 SPLAs. The general information of all patients were listed in Table 2. The general information included gender, age, location, and pathology. The variables of gender (*p* = 0.279) and age (*p* = 0.575) had no statistical significance, while the variables of location (*p* < 0.05) and pathology (*p* < 0.05) showed the significant difference. The tumors of SPLAs were more likely to locate in the right lung than MPLAs (62.2% vs. 60.1%). The pathological type of MPLAs was easier to be MIA (45.9% vs. 32.7%), while that of SPLAs was more prone to be IAC (67.3% vs. 54.1%).

**Table 2:** Patients’ general information

	MPLAs (*n* = 268)	SPLAs (*n* = 826)	*p*
Gender			0.279
Female (%)	168 (62.7%)	487 (59.0%)	
Male (%)	100 (37.3%)	339 (41.0%)	
Age (mean ± standard)	57.4 ± 10.6	56.9 ± 12.9	0.575
Location			< 0.05
Right lung	161 (60.1%)	514 (62.2%)	
Superior lobe	76 (28.4%)	298 (36.1%)	
Middle lobe	40 (14.9%)	54 (6.5%)	
Inferior lobe	45 (16.8%)	162 (19.6%)	
Left lung	107 (39.9%)	312(37.8%)	
Superior lobe	71 (26.5%)	199 (24.1%)	
Inferior lobe	36 (13.4%)	113 (13.7%)	
Pathology			< 0.05
MIA	123 (45.9%)	270 (32.7%)	
IAC	145 (54.1%)	556 (67.3%)	

### Radiomics analysis

**Fig. 3:**
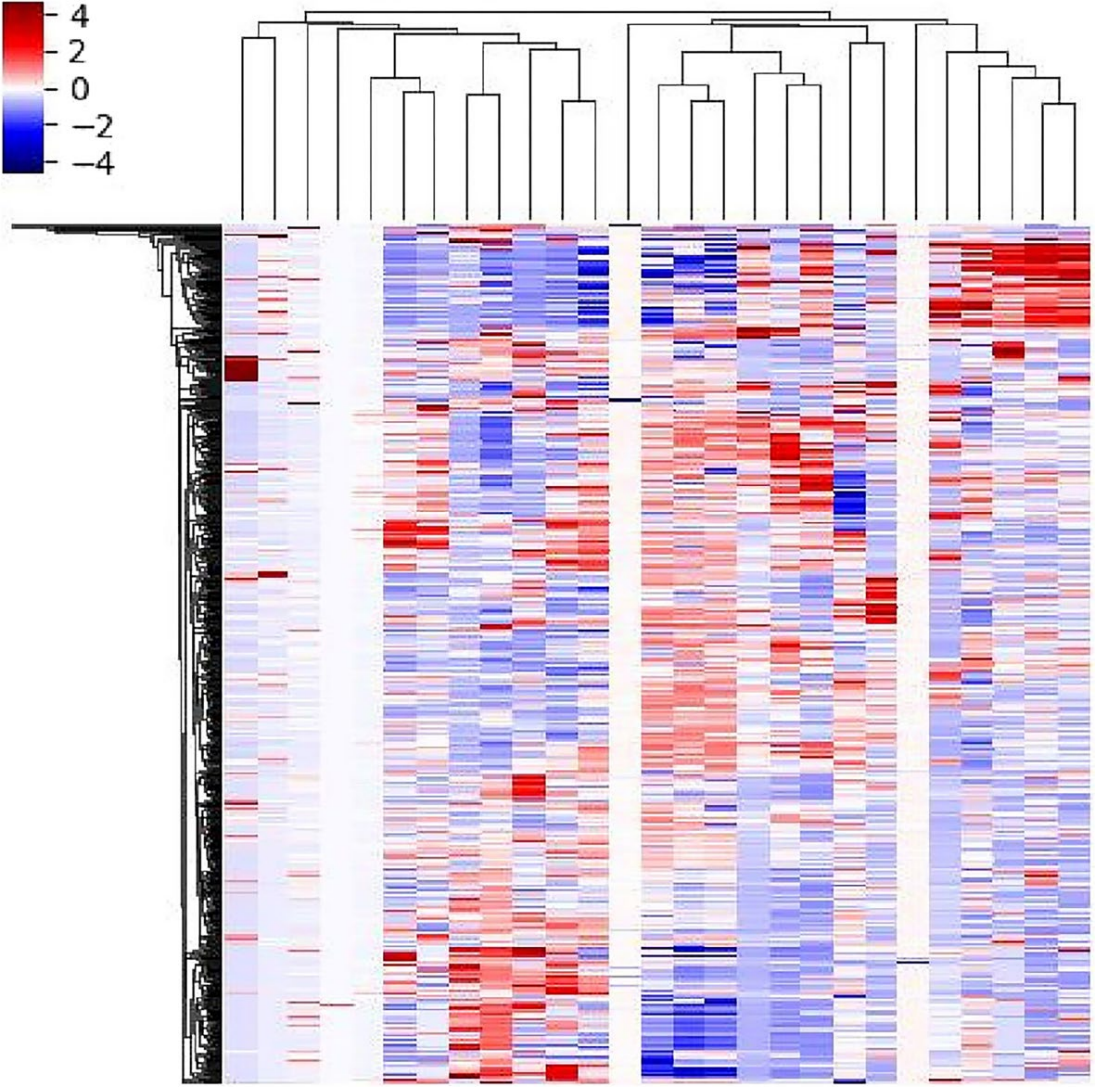
The heatmap of radiomics analysis after feature selection of GBDT, and there were 27 radiomic features selected

The radiomic features between the patients of MPLAs and SPLAs were analyzed. There were 27 radiomic features remained after feature selection of GBDT (Fig. 3) and the machine learning based classifiers including Bayes, forest, k-nearest neighbor, logistic regression, support vector machine, and decision tree were constructed in the training set and confirmed in the testing set. The forest classifier of radiomics (FC-radio) with minimal RSD of 1.82 was chosen for further analysis (Supplementary Material, Table 1). The specific AUC values of six machine-learning algorithms from 100 Bootstrap replication in the training set were listed in Supplementary Material, Table 2. The AUC of this FC-radio in the training set was 0.840 (95%CI, 0.810–0.867) and that of the testing set was 0.670 (95%CI, 0.611–0.724). The low discrimination efficiency of this model indicates that the radiomic difference between tumors of SPLAs and MPLAs was inconspicuous.

### Delta-radiomics analysis

Regardless of the poor efficiency of the FC-radio in distinguishing MPLAs and SPLAs, a further forest machine learning algorithm of delta-radiomics (FC-delta) was conducted. Depending on the different duration of follow-up, we divided the patients into three groups: group A with a follow-up intervals of 3–12 months (105 MPLAs vs. 145 SPLAs), group B with a follow-up intervals of 13–24 months (68 MPLAs vs. 96 SPLAs), and group C with a follow-up intervals of 25–48 months (52 MPLAs vs. 79 SPLAs).

**Fig. 4:**
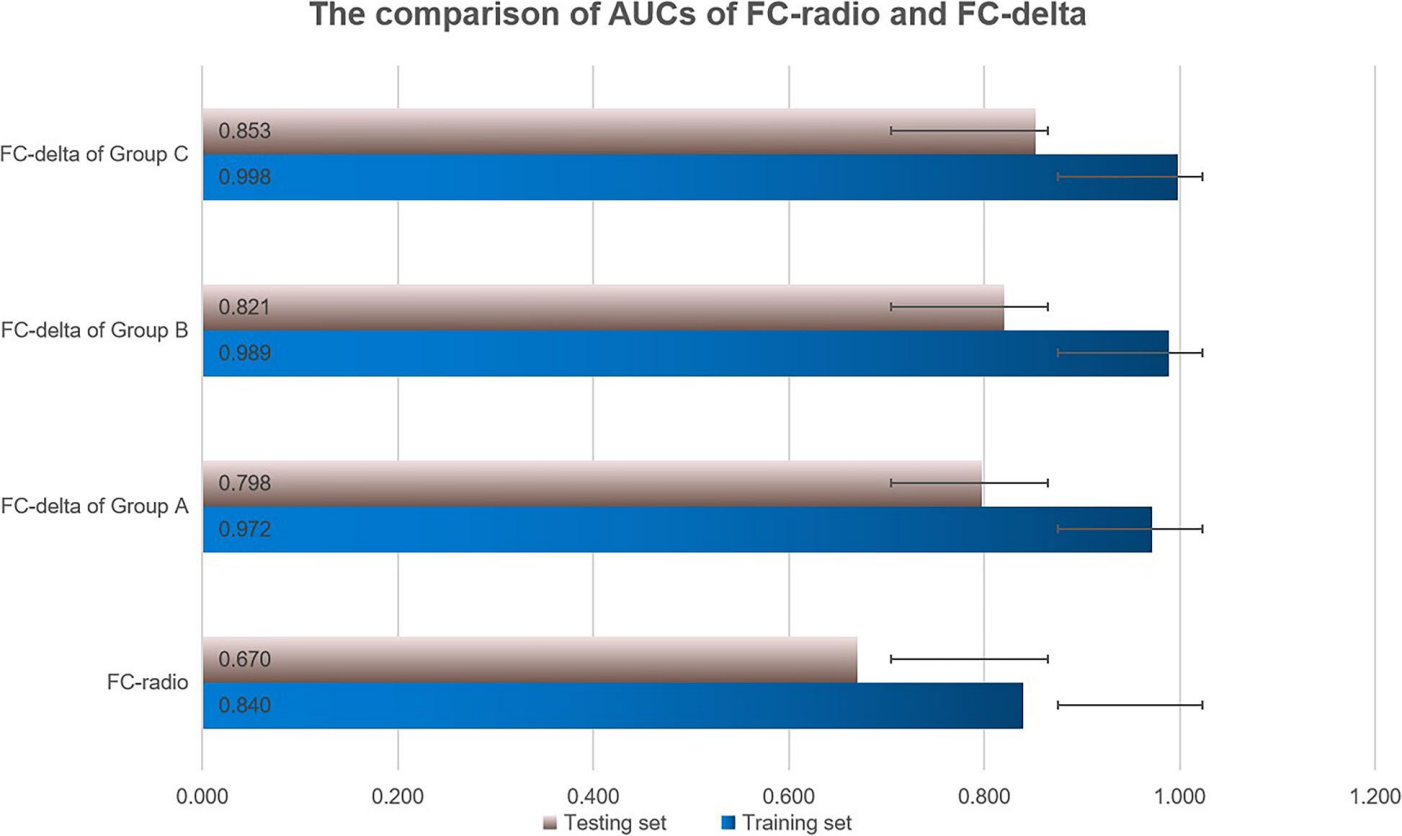
The comparison of AUCs of FC-radio and FC-delta of Group A, B, and C in the training and testing set

The AUC of FC-delta in group A was 0.972 (95%CI, 0.951–0.989) in the training set and was 0.798 (95%CI, 0.704–0.892) in the testing set. The AUC of FC-delta in group B was 0.989 (95%CI, 0.978–0.997) in the training set and was 0.821 (95%CI, 0.708–0.915) in the testing set. The AUC of FC-delta in group C was 0.998 (95%CI, 0.993–1.000) in the training set and was 0.853 (95%CI, 0.752–0.940) in the testing set. With the extension of follow-up intervals, the difference between MPLAs and SPLAs was more obvious (Fig. 4).

## Discussions

We reported a single-institution experience on radiomic differentiation on MPLAs from SPLAs, especially emphasis on their long-term variation. The reported incidence of synchronous lung carcinoma is variably between 0.2 to 20% [13]. In our study, the incidence of MPLAs was 24.5%, which was slightly higher than the reported incidence. It may be related to the universality of chest computed tomography screening programs. There was no statistical difference between the characteristics of gender and age. Slightly different from the outcome in the past study, female gender and smoke free statue were more frequent in multiple primary lung carcinomas [7]. And the MPLAs were easier to the pathological type of MIA compared with SPLAs (45.9% vs. 32.7%, *p* < 0.05). This result supported the previous view that the proportion of MIA and adenocarcinoma in situ is high in synchronous multiple primary lung carcinomas [14]. This also may be due to timely detection of MPLAs at an early stage with regular surveillance for the first primary carcinoma [15]. However, present clinical and traditional radiological methods are unable to understand the different evolution between MPLAs and SPLAs. Thus, studies of novel factors that differ significantly between patients with MPLAs and SPLAs are necessary vehicles for identifying subtleties in two diseases.

We used the method of manual segmentation to delineate tumoral VOI for radiomic analysis. Comparing with automatic and semi-automatic segmentation, manual segmentation carried a certain amount of subjectivity. Therefore the radiomic features greater than 0.75 were selected as robust features and the their mean values from two radiologists were taken for further analysis. It has been reported that radiomics features with a high ICC for volunteer can be considered candidates for radiomics studies [16]. Though deep learning-based segmentation helped to address the inherent limitations of manual segmentation, all relevant radiomics-based models presented similar performance between the two segmentation manners [12]. Patients with first primary cancer are still at risk of developing a secondary tumor at a distant site through metastasis via the lymphatic or circulatory system [17]. Second primary carcinomas showed specific associations with the first one and their nature course were not the same [18]. The etiology of multiple primary lung carcinomas is ambiguous [19]. Our radiomics analysis between MPLAs and SPLAs found of interest that radiomics could identify the difference between two groups with the AUC of 0.840 (95%CI, 0.810–0.867) in the training set and that of 0.670 (95%CI, 0.611–0.724) in the testing set. Nevertheless, this low discrimination efficiency was insufficient to supplied accurate information to better understand the difference between MPLAs and SPLAs. To best of our knowledge, it is the first article focused on the distinction between MPLAs and SPLAs from the point view of radiomics and delta-radiomics.

The crucial challenge regarding MPLAs is what they differed with SPLAs in the course of development, on which both the treatment strategies and prognosis are based [20]. The possible of difference between MPLAs and SPLAs should always be considered during the follow-up surveillance, which determines the subsequent management strategy [21]. It has conclusively been suggested that the overall survival of MPLAs was better than SPLAs with intrapulmonary metastasis [22]. Previous studies have revealed that the large tumor size, lymph node involvement, and the presence of different histological types were independent factors for worse survival [23]. Therefore, a delta-radiomics approach studied the variation of radiomic features during baseline examination and follow-up duration [24]. The AUC of FC-delta of group C was the highest both in the training set (0.998 vs. 0.989 and 0.972) and the testing set (0.853 vs. 0.821 and 0.798). With the extension of follow-up intervals, the difference between MPLAs and SPLAs was more obvious. The literature on survival difference between synchronous MPLAs and SPLAs has quantified and highlighted that the prognosis of synchronous MPLAs was poorer and resembled that of SPLAs of a higher stage [25]. while, the surgical results for multiple primary lung cancer were compatible and acceptable with those for solitary primary lung cancer even with similar histologic subtyping, instead of T4 or M1 stages in the current TNM classification system [26]. Our results suggested that the nature course of two diseases was inconsistent and the delta-radiomics could better distinct the MPLAs and SPLAs than radiomics. We firstly reported the difference of two diseases in terms of both radiomics and delta-radiomics to help us make decision on individual therapy and predict the prognosis of diseases.

Our present study has several limitations. Currently, there are no definitive guideline for the diagnosis and treatment of MPLAs. In 2003, the American College of Chest Physicians (ACCP) developed a new diagnostic criteria for multiple primary lung carcinomas with evaluations of lymphatic and systemic metastasis and the interval between metachronous multiple primary lung carcinomas was extended to at least 4 years [27]. Antakli et al. revised the criteria of Martini and Melamed by adding DNA ploidy validation for distinction [28]. However, they have not widely applied to clinical practice due to its disadvantages of expensive, time consuming, and low sensitivity. Hence, we adopted the most cited criteria of the 2^nd^ edition of ACCP in our research. Second, the MPLAs can be subdivided into synchronous and metachronous phenotypes. Due to the limitation of incidence and sample size, we performed a general analysis of MPLAs which may lead to a biased result. Third, we only enrolled the cohort with pathological types of MIA and IAC to analysis and neglected other pathological types of lung carcinomas. The multiple and solitary primary lung carcinomas with pathological types of adenocarcinoma in situ, squamous carcinoma [29], and so on should further be studied after collecting enough cases. Four, we manually segmented the tumoral VOI to calculate radiomic features. Semi-automatic or automatic segmentation methods for CT images should be carried out in future research.

## Conclusion

In conclusion, our study revealed that the approaches of radiomics and delta-radiomics help to differentiate MPLAs and SPLAs. The radiomic difference between SPLAs and MPLAs was faint and the delta-radiomics better differentiate these patients. Moreover, with the extension of follow-up duration, the delta-radiomics difference between SPLAs and MPLAs appeared more distinctly.

## Supplementary Information

**Additional file 1.**

## Data Availability

The datasets used and analyzed in this article is available from the corresponding author on reasonable request. The code used in this study is available at GitHub (https://github.com/mayq1988/GGN).
