# Supplementary material for: The CT delta-radiomics based machine learning approach in evaluating multiple primary lung adenocarcinoma
Source: BMC Cancer. 2022 Sep 3;22:949. doi: 10.1186/s12885-022-10036-1 (PMC9440600; doi:10.1186/s12885-022-10036-1)
Supplement: Supplementary file 1 — Additional file 1. [file 12885_2022_10036_MOESM1_ESM.doc]

**Supplementary Materials**

1. **Radiomics features**

The radiomics features includes histogram parameters, texture parameters, Gray level co-occurrence matrice (GLCM) parameters, gray level run-length matrice (GLRLM) parameters, and gray level Size Zone Matrice (GLZSM) parameters.

Histogram parameters: It concerns with properties of individual pixels. They describe the distribution of voxel intensities within the CT image through commonly used and basic metrics. It covers the followed parameters: Energy, Entropy, MaxIntensity, MinIntensity, Mean Value, Mean absolute deviation, MedianIntensity, Range ,Root mean square (RMS), Standard deviation (stdDeviation), Uniformity, Variance, Volume Count, Voxel Value Sum, Relative Deviation, Frequency Size, Quantiles, Percentiles, Skewness, and Kurtosis.

Texture parameters: It is one of the important characteristics used in identifying objects or regions of interest in an image, texture represents the appearance of the surface and how its elements are distributed. It is considered an important concept in machine vision, in a sense it assists in predicting the feeling of the surface (e.g. smoothness, coarseness …etc.) from image. It covers the followed parameters: Energy, Entropy, Correlation, Inertia, Cluster Shade, Cluster Prominence.

Form Factor parameters: It includes descriptors of the three-dimensional size and shape of the tumor region. It covered the followed parameters: Sphericity, Surface area, Compactness 1, Compactness 2, Inertia, Cluster Shade, Cluster Prominence.

GLCM parameters: It represents the joint probability of certain sets of pixels having certain gray level values. It calculates how many times a pixel with gray level i occurs jointly with another pixel having a gray value j. By varying the displacement vector d between each pair of pixels. It covers the followed parameters: Energy of GLCM, Entropy of GLCM, Inertia of GLCM, Correlation, Inverse Difference Moment, Haralick features. And the haralick features includes Haralick Correlation, Angular Second Moment, Contrast, Haralick Entropy, Hara Variance, sum Average, sum Variance, sum Entropy, difference Variance, difference Entropy, inverse Difference Moment.

GLRLM parameters: It is defined as the numbers of runs with pixels of gray level i and run length j for a given direction θ. RLMs is generated for each sample image segment having directions (0°, 45°, 90°&135°). It covers the followed parameters: Short/Long Run Emphasis, Gray Level Non-uniformity, Run Length Non-uniformity, Low/High Gray Level Run Emphasis, Short Run Low/High Gray Level Emphasis, Long Run Low/High Gray Level Emphasis.

GLZSM parameters: It is the starting point of Thibault matrices. For a texture image f with N gray levels, it is denoted GSf (s, g) and provides a statistical representation by the estimation of a bi-variate conditional probability density function of the image distribution values. It covers the followed parameters: Small/Large Zone Emphasis, Gray-level Non-uniformity, Zone-Size Non-uniformity, Zone Percentage, Low/High Gray-Level Zone Emphasis, Small Zone Low/High Gray-Level Emphasis, Large Zone Low/High Gray-Level Emphasis, Gray-Level Variance, Zone-Size Variance.

1. **The preprocess of radiomics signature**

The preprocess of radiomics signature comprises the following steps: Image preprocessing, VOI segmentation, and intraclass agreement (ICC) analysis. Image preprocessing: We resampled all the images into a 1.0mm*1.0mm*1.0mm voxel size at X/Y/Z-spacing. Then denoising by Gaussian and normalizing image grey level to a scale from 1 to 32 were automatically proceed in software of AK version 3.0.0 (GE Healthcare). VOI segmentaion: 3D volume of interest (VOI) were delineated by two radiologists with 10 and 12 years of experience independently, in ITK-SNAP software version 3.4.0. The manual defined smooth curve VOI was delineated the tumor margin. Finally, the radiomic features were calculated on AK software, automatically. The intraclass agreement (ICC) analysis: The intra-observer agreements of feature extraction were evaluated by the value of ICC. Intra-observer ICC was computed by comparing extractions of reader A (with 10 years’ experience on abdominal CT) and reader B (with 12 years’ experience on abdominal CT). When the ICC was greater than 0.75, it was considered as good agreement and favorable extraction reproducibility. And the mean value of radiomics features from two radiologists were calculated as robust features for further analysis.

1. **Radiomic feature selection**

Before analyses, variables with zero variance were excluded from analyses. Then, the outlier values were replaced by the median. Finally, the data were standardized by the standardization. Standardization: Extracted texture features were standardized, which removed the unit limits of the data of each feature and converted it into a dimensionless pure value. This allowed the indexes of different units or orders to be compared and weighted. We used a z-score normalization to make the image intensities fit a standard normal distribution with$\mu\text{=0}$and$\sigma\text{=1}$, where$\mu$is the mean value of the images, and$\sigma$is the standard deviation. The normalized values (also called z-scores) of the image intensities (*x*) were calculated as follow:

$$z=\frac{x-\mu}{\sigma}$$

The process of feature selection included univariate analysis and multivariate logistic analysis with stepwise selection method. Firstly, analysis of variance on the extracted features was performed. The variance value is the average of the square of the difference between the value of each variable and the mean. It is the most important method for measuring the dispersion of numeric data. The larger the variance, the greater the fluctuation of the data, and vice versa. So, it is necessary to preferentially eliminate features with a variance of 0 or less. In this study, the variance of each feature was calculated, and then the features greater than the threshold 1 were retained. Second, the correlation test was calculated to reduce data redundancy. The software calculated the paired correlation between each two of the features. If the Spearman correlation coefficient was greater than 0.7, which showed that the two features were highly correlated, one of them was removed. Third, the method of gradient boosting decision tree (GBDT) was used to select radiomic features. Gradient boosting decision tree (GBDT) is a Boosting algorithm based on decision tree as base learner. It builds a decision tree in each iteration to reduce the residual of the current model in the gradient direction. GBDT is commonly used for regression, classification and feature selection. GBDT’s advantages include: (a) It flexible processes of various types of data, including both continuous and discrete data set; (b) It has powerful predictive ability and generalization ability; (c) It has good interpretability, and robustness, can automatically discover high-order relationships between features, and does not require data normalization and other processing^[1]^.

1. **The selection of Machine-learning algorithm**

RSD is the absolute value of the coefficient of variation and is often expressed as a percentage. The equation of RSD was:100 %, σAUC and µ AUC are the standard deviation and mean of the 100 AUC values from 100 Bootstrap replication in the training set, respectively. The RSD (mean±SD) of Bayes, forest, k-nearest neighbor (knn), logistic regression, support vector machine (svm), and decision tree in the training set were calculated, respectively (**Table 1**). The all AUC values of six machine-learning algorithms from 100 Bootstrap replication in the training set were listed in **Table 2**. Therefore, the forest machine learning algorithm with a minimal RSD value was chosen as the most stable algorithm for developing classifiers in distinguishing MPLCs from SPLCs.

**Table 1. The RSD of machine-learning algorithms**

|  | mean | SD | RSD |
| --- | --- | --- | --- |
| Bayes | 0.6451 | 0.02022 | 3.1344 |
| forest | 0.8708 | 0.01585 | 1.8202 |
| knn | 0.7561 | 0.01811 | 2.3952 |
| logistic | 0.6936 | 0.01873 | 2.7004 |
| svm | 0.6204 | 0.03901 | 6.2879 |
| tree | 0.7650 | 0.03488 | 4.5595 |

**Table 2. The AUCs of machine-learning algorithms from 100 Bootstrap replication**

| algorithm | Bayes | forest | knn | logistic | svm | tree |
| --- | --- | --- | --- | --- | --- | --- |
| 1 | 0.639 | 0.879 | 0.736 | 0.685 | 0.537 | 0.748 |
| 2 | 0.648 | 0.869 | 0.78 | 0.714 | 0.588 | 0.798 |
| 3 | 0.646 | 0.849 | 0.776 | 0.734 | 0.694 | 0.673 |
| 4 | 0.66 | 0.858 | 0.745 | 0.706 | 0.679 | 0.798 |
| 5 | 0.638 | 0.856 | 0.743 | 0.672 | 0.607 | 0.795 |
| 6 | 0.668 | 0.871 | 0.714 | 0.692 | 0.608 | 0.813 |
| 7 | 0.666 | 0.869 | 0.751 | 0.701 | 0.668 | 0.767 |
| 8 | 0.664 | 0.871 | 0.765 | 0.709 | 0.665 | 0.794 |
| 9 | 0.643 | 0.875 | 0.781 | 0.707 | 0.658 | 0.768 |
| 10 | 0.623 | 0.84 | 0.721 | 0.692 | 0.63 | 0.755 |
| 11 | 0.658 | 0.85 | 0.771 | 0.69 | 0.618 | 0.743 |
| 12 | 0.649 | 0.879 | 0.752 | 0.721 | 0.671 | 0.768 |
| 13 | 0.667 | 0.876 | 0.744 | 0.692 | 0.609 | 0.749 |
| 14 | 0.613 | 0.892 | 0.738 | 0.662 | 0.569 | 0.82 |
| 15 | 0.674 | 0.883 | 0.78 | 0.692 | 0.618 | 0.779 |
| 16 | 0.647 | 0.877 | 0.786 | 0.697 | 0.609 | 0.787 |
| 17 | 0.668 | 0.861 | 0.767 | 0.711 | 0.678 | 0.748 |
| 18 | 0.607 | 0.865 | 0.743 | 0.676 | 0.597 | 0.774 |
| 19 | 0.664 | 0.888 | 0.759 | 0.7 | 0.604 | 0.771 |
| 20 | 0.667 | 0.867 | 0.776 | 0.705 | 0.637 | 0.786 |
| 21 | 0.643 | 0.859 | 0.749 | 0.68 | 0.573 | 0.755 |
| 22 | 0.657 | 0.879 | 0.753 | 0.678 | 0.613 | 0.755 |
| 23 | 0.638 | 0.87 | 0.771 | 0.707 | 0.665 | 0.764 |
| 24 | 0.669 | 0.857 | 0.744 | 0.705 | 0.659 | 0.792 |
| 25 | 0.627 | 0.875 | 0.744 | 0.694 | 0.628 | 0.754 |
| 26 | 0.66 | 0.889 | 0.751 | 0.678 | 0.627 | 0.745 |
| 27 | 0.661 | 0.864 | 0.748 | 0.712 | 0.628 | 0.79 |
| 28 | 0.627 | 0.891 | 0.712 | 0.669 | 0.64 | 0.732 |
| 29 | 0.66 | 0.871 | 0.742 | 0.704 | 0.655 | 0.772 |
| 30 | 0.601 | 0.871 | 0.747 | 0.666 | 0.55 | 0.755 |
| 31 | 0.63 | 0.853 | 0.738 | 0.685 | 0.618 | 0.776 |
| 32 | 0.638 | 0.895 | 0.749 | 0.704 | 0.533 | 0.769 |
| 33 | 0.631 | 0.857 | 0.76 | 0.69 | 0.658 | 0.722 |
| 34 | 0.626 | 0.883 | 0.766 | 0.678 | 0.641 | 0.735 |
| 35 | 0.675 | 0.882 | 0.779 | 0.74 | 0.719 | 0.754 |
| 36 | 0.649 | 0.887 | 0.772 | 0.703 | 0.608 | 0.808 |
| 37 | 0.633 | 0.865 | 0.774 | 0.675 | 0.594 | 0.786 |
| 38 | 0.622 | 0.877 | 0.725 | 0.663 | 0.605 | 0.752 |
| 39 | 0.667 | 0.897 | 0.791 | 0.717 | 0.676 | 0.806 |
| 40 | 0.654 | 0.871 | 0.777 | 0.692 | 0.633 | 0.776 |
| 41 | 0.631 | 0.884 | 0.743 | 0.682 | 0.57 | 0.793 |
| 42 | 0.677 | 0.89 | 0.741 | 0.7 | 0.624 | 0.806 |
| 43 | 0.656 | 0.871 | 0.732 | 0.668 | 0.627 | 0.753 |
| 44 | 0.672 | 0.857 | 0.766 | 0.708 | 0.607 | 0.739 |
| 45 | 0.621 | 0.855 | 0.74 | 0.675 | 0.622 | 0.751 |
| 46 | 0.662 | 0.87 | 0.756 | 0.726 | 0.651 | 0.748 |
| 47 | 0.654 | 0.853 | 0.736 | 0.69 | 0.609 | 0.734 |
| 48 | 0.681 | 0.87 | 0.759 | 0.751 | 0.687 | 0.719 |
| 49 | 0.615 | 0.885 | 0.755 | 0.684 | 0.569 | 0.785 |
| 50 | 0.662 | 0.865 | 0.758 | 0.69 | 0.607 | 0.802 |
| 51 | 0.645 | 0.865 | 0.787 | 0.704 | 0.636 | 0.779 |
| 52 | 0.613 | 0.865 | 0.738 | 0.658 | 0.576 | 0.711 |
| 53 | 0.632 | 0.892 | 0.753 | 0.703 | 0.54 | 0.75 |
| 54 | 0.592 | 0.869 | 0.753 | 0.682 | 0.608 | 0.751 |
| 55 | 0.662 | 0.884 | 0.774 | 0.702 | 0.627 | 0.798 |
| 56 | 0.63 | 0.872 | 0.754 | 0.698 | 0.652 | 0.791 |
| 57 | 0.656 | 0.872 | 0.786 | 0.706 | 0.643 | 0.73 |
| 58 | 0.654 | 0.876 | 0.762 | 0.699 | 0.612 | 0.716 |
| 59 | 0.653 | 0.881 | 0.766 | 0.703 | 0.647 | 0.78 |
| 60 | 0.621 | 0.856 | 0.756 | 0.67 | 0.632 | 0.762 |
| 61 | 0.648 | 0.835 | 0.769 | 0.665 | 0.583 | 0.68 |
| 62 | 0.62 | 0.86 | 0.762 | 0.679 | 0.538 | 0.678 |
| 63 | 0.619 | 0.864 | 0.736 | 0.664 | 0.598 | 0.822 |
| 64 | 0.654 | 0.851 | 0.756 | 0.685 | 0.627 | 0.784 |
| 65 | 0.661 | 0.896 | 0.779 | 0.706 | 0.636 | 0.767 |
| 66 | 0.618 | 0.905 | 0.755 | 0.679 | 0.598 | 0.774 |
| 67 | 0.63 | 0.867 | 0.774 | 0.685 | 0.644 | 0.724 |
| 68 | 0.647 | 0.865 | 0.772 | 0.712 | 0.668 | 0.769 |
| 69 | 0.623 | 0.894 | 0.768 | 0.664 | 0.538 | 0.8 |
| 70 | 0.659 | 0.871 | 0.761 | 0.71 | 0.661 | 0.75 |
| 71 | 0.634 | 0.867 | 0.734 | 0.703 | 0.645 | 0.824 |
| 72 | 0.663 | 0.849 | 0.755 | 0.703 | 0.649 | 0.778 |
| 73 | 0.63 | 0.875 | 0.795 | 0.688 | 0.615 | 0.758 |
| 74 | 0.613 | 0.894 | 0.738 | 0.682 | 0.619 | 0.797 |
| 75 | 0.683 | 0.87 | 0.78 | 0.714 | 0.671 | 0.809 |
| 76 | 0.676 | 0.851 | 0.762 | 0.706 | 0.669 | 0.831 |
| 77 | 0.64 | 0.886 | 0.722 | 0.678 | 0.589 | 0.669 |
| 78 | 0.652 | 0.878 | 0.738 | 0.681 | 0.558 | 0.802 |
| 79 | 0.646 | 0.833 | 0.773 | 0.694 | 0.62 | 0.79 |
| 80 | 0.664 | 0.873 | 0.766 | 0.702 | 0.64 | 0.736 |
| 81 | 0.63 | 0.844 | 0.734 | 0.695 | 0.654 | 0.734 |
| 82 | 0.654 | 0.893 | 0.743 | 0.693 | 0.638 | 0.817 |
| 83 | 0.615 | 0.873 | 0.725 | 0.68 | 0.609 | 0.786 |
| 84 | 0.637 | 0.84 | 0.76 | 0.683 | 0.641 | 0.763 |
| 85 | 0.626 | 0.886 | 0.781 | 0.726 | 0.675 | 0.787 |
| 86 | 0.655 | 0.87 | 0.767 | 0.702 | 0.645 | 0.738 |
| 87 | 0.678 | 0.897 | 0.759 | 0.716 | 0.569 | 0.815 |
| 88 | 0.621 | 0.895 | 0.746 | 0.698 | 0.623 | 0.749 |
| 89 | 0.63 | 0.863 | 0.739 | 0.682 | 0.575 | 0.708 |
| 90 | 0.634 | 0.849 | 0.753 | 0.679 | 0.601 | 0.729 |
| 91 | 0.679 | 0.879 | 0.793 | 0.708 | 0.646 | 0.792 |
| 92 | 0.636 | 0.834 | 0.753 | 0.648 | 0.588 | 0.747 |
| 93 | 0.659 | 0.884 | 0.771 | 0.696 | 0.516 | 0.793 |
| 94 | 0.632 | 0.858 | 0.743 | 0.675 | 0.634 | 0.673 |
| 95 | 0.672 | 0.895 | 0.76 | 0.72 | 0.628 | 0.811 |
| 96 | 0.626 | 0.848 | 0.773 | 0.667 | 0.563 | 0.742 |
| 97 | 0.636 | 0.895 | 0.74 | 0.691 | 0.613 | 0.786 |
| 98 | 0.659 | 0.87 | 0.747 | 0.725 | 0.607 | 0.739 |
| 99 | 0.626 | 0.865 | 0.73 | 0.672 | 0.619 | 0.767 |
| 100 | 0.658 | 0.86 | 0.767 | 0.707 | 0.619 | 0.748 |

1. **The evaluations of all forest classifiers**

To the radiomics based forest machine learning classifier, there were 27 radiomic features included and the predictive parameters in both the training and validation set were as follows:

| Item | Train | Validation |
| --- | --- | --- |
| Accuracy | 0.8 | 0.775 |
| f1_score | 0.314 | 0.229 |
| Recall | 0.187 | 0.136 |
| Precision | 0.972 | 0.733 |
| AUC | 0.84 (0.81, 0.867) | 0.67 (0.611, 0.724) |
| Sensitivity | 0.187 | 0.136 |
| Specificity | 0.998 | 0.984 |
| positive prediction | 0.972 | 0.733 |
| negative prediction | 0.791 | 0.777 |
| positive llr | 108.182 | 8.42 |
| negatice llr | 0.814 | 0.878 |

To the delta-radiomics based forest machine learning classifier in group A (3-12 months), there were 23 delta-radiomic features included and the predictive parameters in both the training and validation set were as follows:

| Item | Train | Validation |
| --- | --- | --- |
| Accuracy | 0.931 | 0.747 |
| f1_score | 0.914 | 0.667 |
| Recall | 0.877 | 0.594 |
| Precision | 0.955 | 0.76 |
| AUC | 0.972 (0.951, 0.989) | 0.798 (0.704, 0.892) |
| Sensitivity | 0.877 | 0.594 |
| Specificity | 0.971 | 0.86 |
| positive prediction | 0.955 | 0.76 |
| negative prediction | 0.917 | 0.74 |
| positive llr | 29.808 | 4.255 |
| negatice llr | 0.127 | 0.472 |

To the delta-radiomics based forest machine learning classifier in group B (13-24 months), there were 22 delta-radiomic features included and the predictive parameters in both the training and validation set were as follows:

| Item | Train | Validation |
| --- | --- | --- |
| Accuracy | 0.921 | 0.7 |
| f1_score | 0.899 | 0.545 |
| Recall | 0.851 | 0.429 |
| Precision | 0.952 | 0.75 |
| AUC | 0.989 (0.978, 0.997) | 0.821 (0.708, 0.915) |
| Sensitivity | 0.851 | 0.429 |
| Specificity | 0.97 | 0.897 |
| positive prediction | 0.952 | 0.75 |
| negative prediction | 0.903 | 0.684 |
| positive llr | 28.511 | 4.143 |
| negatice llr | 0.154 | 0.637 |

To the delta-radiomics based forest machine learning classifier in group C (25-48 months), there were 22 delta-radiomic features included and the predictive parameters in both the training and validation set were as follows:

| Item | Train | Validation |
| --- | --- | --- |
| Accuracy | 0.989 | 0.7 |
| f1_score | 0.986 | 0.714 |
| Recall | 0.972 | 0.938 |
| Precision | 1.0 | 0.577 |
| AUC | 0.998 (0.993, 1.0) | 0.853 (0.752, 0.94) |
| Sensitivity | 0.972 | 0.938 |
| Specificity | 1.0 | 0.542 |
| positive prediction | 1.0 | 0.577 |
| negative prediction | 0.982 | 0.929 |
| positive llr | inf | 2.045 |
| negatice llr | 0.028 | 0.115 |

1. **Reference**

1. Liang Y, Zhang S, Qiao H, Cheng Y. iEnhancer-MFGBDT: Identifying enhancers and their strength by fusing multiple features and gradient boosting decision tree. Math Biosci Eng. 2021 Oct 14;18(6):8797-8814. doi: 10.3934/mbe.2021434. PMID: 34814323.
